# *In vitro* characterization of neurite extension using induced pluripotent stem cells derived from lissencephaly patients with *TUBA1A* missense mutations

**DOI:** 10.1186/s13041-016-0246-y

**Published:** 2016-07-19

**Authors:** Yohei Bamba, Tomoko Shofuda, Mitsuhiro Kato, Ritsuko K. Pooh, Yoko Tateishi, Jun-ichi Takanashi, Hidetsuna Utsunomiya, Miho Sumida, Daisuke Kanematsu, Hiroshi Suemizu, Yuichiro Higuchi, Wado Akamatsu, Denis Gallagher, Freda D. Miller, Mami Yamasaki, Yonehiro Kanemura, Hideyuki Okano

**Affiliations:** Department of Physiology, Keio University School of Medicine, 35 Shinanomachi, Shinjuku-ku, Tokyo 160-8582 Japan; Division of Regenerative Medicine, Institute for Clinical Research, Osaka National Hospital, National Hospital Organization, 2-1-14 Hoenzaka, Chuo-ku, Osaka 540-0006 Japan; Division of Stem Cell Research, Institute for Clinical Research, Osaka National Hospital, National Hospital Organization, Osaka, Japan; Department of Pediatrics, Yamagata University Faculty of Medicine, 2-2-2 Iida-Nishi, Yamagata-shi, Yamagata 990-9585 Japan; CRIFM Clinical Research Institute of Fetal Medicine Pooh Maternity Clinic, 7-1-24 Uehommachi, Tennoji, Osaka 543-0001 Japan; Department of Obstetrics and Gynecology, Okayama Medical Center, National Hospital Organization, 1711-1 Tamasu, Okayama, Japan; Department of Pediatrics, Tokyo Women’s Medical University, Yachiyo Medical Center, 477-96 Owadashinden, Yachiyo-shi, 276-8524 Japan; Department of Radiological Science, International University of Health and Welfare, Graduate School, 2-4-16, Momochihama, Sawara-ku, Fukuoka 814-0001 Japan; Laboratory Animal Research Department, Central Institute for Experimental Animals, 3-25-12 Tonomachi, Kawasaki-ku, Kawasaki, Kanagawa 210-0821 Japan; Program in Neuroscience and Mental Health, The Hospital for Sick Children, Toronto, ON M5G 0A7 Canada; McEwen Center for Regenerative Medicine, University Health Network, Toronto, ON M5G 0A7 Canada; Department of Molecular Genetics, University of Toronto, Toronto, ON M5G1X5 Canada; Department of Physiology, University of Toronto, Toronto, ON M5G 1X5 Canada; Department of Pediatric Neurosurgery, Takatsuki General Hospital, 1-3-13、Kosobe cho, Takatsuki, 569-1192 Japan; Division of Molecular Medicine, Institute for Clinical Research, Osaka National Hospital, National Hospital Organization, Osaka, Japan; Department of Neurosurgery, Osaka National Hospital, National Hospital Organization, Osaka, Japan

**Keywords:** Lissencephaly, Induced pluripotent stem cells, TUBA1A, Neural progenitor cells

## Abstract

**Background:**

Lissencephaly, or smooth brain, is a severe congenital brain malformation that is thought to be associated with impaired neuronal migration during corticogenesis. However, the exact etiology of lissencephaly in humans remains unknown. Research on congenital diseases is limited by the shortage of clinically derived resources, especially for rare pediatric diseases. The research on lissencephaly is further limited because gyration in humans is more evolved than that in model animals such as mice. To overcome these limitations, we generated induced pluripotent stem cells (iPSCs) from the umbilical cord and peripheral blood of two lissencephaly patients with different clinical severities carrying alpha tubulin (*TUBA1A*) missense mutations (Patient A, p.N329S; Patient B, p.R264C).

**Results:**

Neural progenitor cells were generated from these iPSCs (iPSC-NPCs) using SMAD signaling inhibitors. These iPSC-NPCs expressed *TUBA1A* at much higher levels than undifferentiated iPSCs and, like fetal NPCs, readily differentiated into neurons. Using these lissencephaly iPSC-NPCs, we showed that the neurons derived from the iPSCs obtained from Patient A but not those obtained from Patient B showed abnormal neurite extension, which correlated with the pathological severity in the brains of the patients.

**Conclusion:**

We established iPSCs derived from lissencephaly patients and successfully modeled one aspect of the pathogenesis of lissencephaly *in vitro* using iPSC-NPCs and iPSC-derived neurons. The iPSCs from patients with brain malformation diseases helped us understand the mechanism underlying rare diseases and human corticogenesis without the use of postmortem brains.

**Electronic supplementary material:**

The online version of this article (doi:10.1186/s13041-016-0246-y) contains supplementary material, which is available to authorized users.

## Background

Dysfunction in the complicated cellular dynamics involved in the development of the cerebral cortex causes various congenital brain malformations during different stages of the development of the central nervous system (CNS) [1]. The two major modes of development in the human cerebral cortex are tangential and radial expansion. Tangential expansion evolves as the number of progenitor cells increases, as observed recently in the outer subventricular zone radial glia of gyrencephalic species including humans [2, 3]. A decreased number or abnormal division of progenitor cells leads to microcephaly, or small brain. Radial expansion depends on neuronal migration in a basal to apical direction along the processes of the radial glia, and dysfunction in this process impairs corticogenesis in lissencephaly.

Lissencephaly, with disturbed cerebral cortical lamination, is one of the most severe brain malformations. In lissencephaly, the surface of the brain appears smooth owing to insufficient gyration, which is why this condition is called smooth brain. Lissencephaly is mainly caused by mutations in genes, many of which are involved in microtubule function. In addition to the genes encoding microtubule-associated proteins, alpha tubulin (*TUBA1A*) mutations have recently been shown to cause abnormal neuronal migration [4]. In humans, *TUBA1A* mutations have been identified in lissencephaly patients whose brains showed a smooth surface owing to severely impaired lamination of the cerebral cortex [4–6]. Lissencephaly in humans is an extremely rare disease, and lissencephaly patients often die within a few years, thus making it difficult to obtain viable patient-derived cells including neurons. This limitation has greatly restricted the complete elucidation of the etiology of lissencephaly in humans.

Therefore, to investigate the pathogenic mechanisms underlying lissencephaly in humans, we established induced pluripotent stem cells (iPSCs) from lissencephaly patients. Using neural progenitor cells and neurons generated from patient-derived iPSCs, we aimed to elucidate the disease pathology and to develop novel therapies.

## Methods

### Generation of iPSCs

**Fig. 1:**
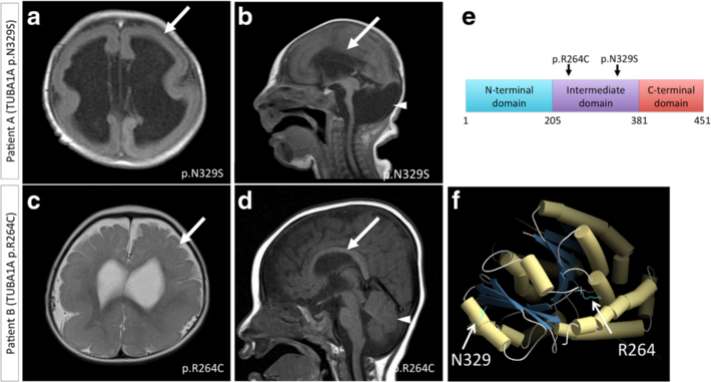
Magnetic resonance imaging (MRI) of two patients. **a**, **b** Patient A (p.N329S *TUBA1A* mutation) shows lissencephaly with cerebellar hypoplasia. Thin cerebral mantle and agyric cerebral cortices (*arrow* in figure **a**) are observed without an anterior-posterior gradient. The corpus callosum is not present (*arrow* in figure **b**). **c**, **d** Patient B (p.R264C *TUBA1A* mutation) shows pachygyria with a posterior-anterior gradient (*arrow* in figure **c**). Cerebellar and brain stem hypoplasia are not as clear as in Patient A (*arrowhead* in figure **d**). The corpus callous is present (*arrow* in figure **d**). **e** Schematic structure of TUBA1A is represented based on a previous report [34]. Both missense mutations in TUBA1A were located in the intermediate domain of TUBA1A. **f** Three-dimensional structure of the TUBA1A protein. Helices are presented as cylinders. The arrows show the residue of each mutation. The p.N329S mutation was located on alpha-helix H10, which formed the interface with beta-tubulin. The p.R264C mutation was located between alpha-helix H8 and the beta sheet, which could be responsible for providing stability to the tertiary structure

Umbilical cords collected from Patient A with the p.N329S *TUBA1A* mutation (Fig. 1) and from vaginally delivered full-term fetal adnexa of healthy volunteers (male) were digested with collagenase I (Life Technologies, Carlsbad, CA, USA) and dispase (Life Technologies) for 30 min at 37 °C. The cells liberated from the tissue were then collected by centrifugation and seeded in T75 flasks in Dulbecco’s modified Eagle’s medium/nutrient mixture F-12 (DMEM/F12) (Sigma-Aldrich, St. Louis, MO, USA) containing 10 % fetal bovine serum (FBS), 15 mM HEPES, and antibiotic-antimycotic solution (Life Technologies) [7]. Umbilical cord-derived stromal cells (UCCs) were passaged after 1 week and used for iPSC generation after 3–5 passages.

Peripheral blood mononuclear cells (PBMCs) from Patient B with the p.R264C *TUBA1A* mutation (Fig. 1) and from a healthy adult volunteer were isolated using Ficoll-Paque (GE Healthcare, Buckinghamshire, UK) according to the manufacturer’s instructions. The isolated PBMCs were activated with immobilized anti-CD3 monoclonal antibodies (Orthoclone OKTR3 Injection, Janssen-Kyowa, Tokyo, Japan) and expanded in soluble interleukin (IL)-2-containing ALyS203 medium (NIPRO, Japan) with 10 % FBS as previously described, with minor modifications [8]. In this study, we used the human iPSC line (201B7) [9] derived from human dermal fibroblasts from the facial dermis of a 36-year-old Caucasian female as the control (obtained from the RIKEN cell bank (Tsukuba, Japan)).

For iPSC generation, reprogramming episomal vectors (see below) were nucleofected using Amaxa Nucleofector I (a Neonatal Fibroblast Nucleofector Kit with the U-020 program for UCCs and a Human T cell Nucleofector Kit with the T-023 program for PBMCs; Lonza, Basel, Switzerland). Plasmids were used with combinations of pCXLE-hOct3/4-shp53, pCXLE-hUL, and pCXLE-hSK [10] for UCCs and PBMCs [11] or pCEB-hSKO and pCEB-hULG for UCCs. All the plasmids were graciously provided by Dr. Keisuke Okita and Prof. Shinya Yamanaka (Kyoto University, Kyoto, Japan). The subsequent procedures, including colony isolation and propagation, were performed as previously described [12]. The generated iPSCs were characterized using immunocytochemistry for the pluripotency-associated transcription factors OCT3/4 and NANOG; quantitative RT-PCR for the marker genes *OCT3/4, SOX2, NANOG, ZFP42,* and *DNMT3B*; and PCR according to protocols provided by the Center for Developmental Biology (RIKEN, http://www.cdb.riken.jp) and the Center for iPS Cell Research and Application (Kyoto University, https://www.cira.kyoto-u.ac.jp) to confirm that the episomal vectors did not persist.

The teratoma formation assay for the iPSCs was performed with subcutaneous transplantations into NOD/Shi-SCID IL2Rγ null (NOG) mice [12, 13] in the Animal Facility of the CIEA (Central Institute for Experimental Animals, Kawasaki, Japan), in accordance with the guidelines of the Animal Care Committee at CIEA, and was approved by the Animal Care Committee at CIEA (No.11029A). The genomic sequences of *TUBA1A* were confirmed using previously reported methods [6].

The human embryonic stem cell line KhES1 [14] was propagated at Keio University in accordance with Japanese guidelines for the utilization of human embryonic stem cells with the approval of the Ministry of Education, Culture, Sports, Science and Technology of Japan and the ethical committee of Keio University. RNA samples from the KhES1 cells were isolated using an RNeasy minikit (QIAGEN, Valencia, CA, USA) and were used as a standard for quantitative RT-PCR. Summaries of the donor information and radiological images are shown in Table 1 and Fig. 1a–d, respectively. Both the *TUBA1A* mutations in these patients were located in the intermediate domain of TUBA1A, as shown in the schematic illustration in Fig. 1e. A karyotyping analysis was performed using conventional Giemsa staining and G-banding in all the generated iPSCs as described previously [12].

**Table 1:** Donor information and radiological findings

Patient	Gender	Age (years)	Diagnosis	LIS grading [35]	Cerebral cortex	Corpus callosum	Cerebellum	Basal ganglia	Brain stem	Clinical information	Same mutation reported previously
A	Male	0	Microlissencephaly with cerebellar hypoplasia	Grade 1	Lissencephaly	Agenesis	Hypoplastic	Hypoplastic	Hypoplastic	Brain stem dysfunction, seizure, and developmental delay. Died at 2 years old.	Kumar RA et al. [5]
B	Male	2	Lissencephaly	Grade 4	Agyria-pachygyria (posterior > anterior gradient)	Present, abnormal	Not hypoplastic	Hypoplastic	Not hypoplastic	Intellectual delay and intractable seizures. Word speech at 3 years old. Has survived for 4 years now.	Keays DA et al. [4]

### Gene and protein analyses

The sequences of the *TUBA1A* gene in this report are based on the sequences obtained from GenBank (Accession NM_006009). The protein structure of TUBA1A [15] was obtained from Protein Data Bank Japan (PDBj: 1JFF), and the illustration was generated using CueMol2 (http:/http://www.cuemol.org/en/).

### Neural induction by dual SMAD inhibition and neurosphere propagation

The highly efficient dual inhibition of SMAD signaling was used for neural induction of the iPSCs [16]. Briefly, the iPSCs cultured in growth-factor-reduced (GFR) Matrigel (BD Biosciences, San Diego, CA, USA)-coated dishes were collected and suspended in low-attachment dishes (Primesurface, Sumitomo Bakelite, Tokyo, Japan) to form embryoid bodies (EBs). For neural induction, the EBs were subsequently cultured in DMEM/F12 containing 5 % B27 supplement (Life Technologies), 5 % N2 supplement (Life Technologies), 20 ng/ml recombinant human (rh) basic fibroblast growth factor (bFGF) (Wako, Osaka, Japan), 10 μM SB431542 (SB; Sigma-Aldrich), and 1 μM dorsomorphin (DSM; Wako) for 2 weeks under 5 % O_2_. To obtain neural progenitor cells (NPCs) as neurospheres, the cell aggregates were mechanically dissociated and cultured in DMEM/F12 containing 2 % B27 supplement (Life Technologies), 20 ng/ml rh-basic fibroblast growth factor (bFGF; PeproTech, Rocky Hill NJ, USA), 20 ng/ml rh-epidermal growth factor (EGF; PeproTech), 10 ng/ml rh-leukemia inhibitory factor (LIF; Millipore, Billerica, MA, USA), and 5 μg/ml heparin (Sigma-Aldrich). The neurospheres were passaged before the center of the spheres darkened, typically every 12–14 days as described previously [17].

### Differentiation of the iPSC-derived neurospheres

At approximately passage six, the neurospheres were plated onto GFR Matrigel (BD)-coated dishes in neurobasal medium (Life Technologies) containing 2 % B27 supplement (Life Technologies) and 2 mM L-glutamine (Life Technologies) (differentiation medium). For the PiggyBac vector transfection experiments, iPSC-derived neurospheres were used at stages beyond six passages when the regional identities could be caudalized and stably propagated. For differentiation in dissociated culture, the neurospheres were dissociated using TrypLE Select (Life technologies) for 5 min at 37 °C, and the dissociated iPSC-NPCs were seeded onto GFR-Matrigel (BD)-coated 96-well plates and 6-well plates in differentiation media.

### Quantitative RT-PCR

RNA was extracted from the aggregates and neurospheres using an RNeasy Mini Kit (QIAGEN) and DNase set (QIAGEN), and cDNAs were synthesized using PrimeStarRT Reagent Kits (Takara Bio, Shiga, Japan). Quantitative PCR was performed using a PowerSYBR Green PCR Mix (Life Technologies) and an ABI7300 Real-time PCR system (Life Technologies) using gene-specific primer pairs (Additional file 1: Table S1). The expression (Ct values) of each gene was normalized to that of an internal control, and the normalized expression was compared using the ΔΔCt method as previously described [18]. As a human telencephalic control, 201B7 iPSC-derived telencephalic SFEB (serum-free floating-culture of embryoid body-like) aggregates at day 25 [19] were used. GDC90 human glial cell lines were used as a human glial cell control [20].

### Visualization of the neurite extension of neurons derived from iPSCs of lissencephaly patients using the glia-supported neurite extension method

We used the human glial cell line GDC90 as a scaffold [20] to support the robust differentiation and neurite extension of the iPSC-derived neurons. To distinguish glial cell lines and iPSC-derived neural progenitor cells (iPSC-NPCs) and to illustrate the morphology of the neurites migrating from each iPSC-NPC-derived young neuron, the CAG promoter-driven [21] PiggyBac vector pPB-CAG-EGFP-neo [20] was nucleofected into the iPSC-derived neurospheres using Amaxa Nucleofector I (Lonza) as previously described [20]. After positive selection using Geneticin at a concentration of 200 μg/ml (Life Technologies), the enhanced green fluorescent protein (EGFP)-labeled iPSC-NPCs were used for further analyses.

To measure the neurites migrating from the neurospheres, iPSC-NPCs (5 × 10^3^ cells or 2.5 × 10^3^ cells for time-lapse imaging) and GDC90s (5 × 10^3^ cells or 7.5 × 10^3^ cells for time-lapse imaging) were seeded and re-aggregated in low-attachment 96-well plates with spindle-shaped bottoms (Sumitomo Bakelite). After 24 h, the re-aggregated spheres were plated onto GFR-Matrigel-coated glass-bottom dishes in differentiation medium. In some experiments requiring the visualization of the glial cell line, tdTomato-labeled GDC90s [20] were used.

To quantify the behavior of the iPSC-NPCs derived from lissencephaly patients, the length of the EGFP-fluorescent neurites extending from the soma of the iPSC-NPCs existing at the edge of the fluorescent clusters was measured using ImageJ software on day 5. Time-lapse images during the differentiation and migration of the control and lissencephaly iPSC-derived neurons were acquired using an IncuCyte ZOOM HD (Essen BioSciences, Ann Arbor, MI, USA) every 4 h. The video files were exported in AVI format at six frames per second.

### Immunocytochemistry and immunohistochemistry

Undifferentiated iPSCs, forebrain-committed neurons, and neurospheres were fixed in 4 % paraformaldehyde and stained using primary and secondary antibodies as described below. The samples were examined using a confocal laser-scanning microscope (LSM510, Carl Zeiss, Hallbergmoos, Germany). The following primary antibodies were used (host, dilution, manufacturer): neuronal class III β-tubulin (TUJ1; mouse, 1:500, Covance, Princeton, NJ), brain lipid-binding protein (BLBP; rabbit, 1:500, Millipore), GFAP (rabbit, 1:80, Sigma-Aldrich), SOX1 (mouse, 1:500, BD Pharmingen), NESTIN (rabbit, 1:500 [22] and mouse, 1:500, Millipore), GFP (mouse, 1:500, Clontech, Palo Alto, CA, USA), doublecortin (DCX; rabbit, 1:500, Cell Signaling), OCT-4 (mouse, 1:200, BD), NANOG (rabbit, 1:200, ReproCELL), TRA-1-60 (mouse, 1:200, Chemicon), SSEA-3 (rat, 1:200, Chemicon), SSEA-4 (mouse, 1:200, Chemicon), and TRA-1-81 (mouse, 1:200, Chemicon). The following secondary antibodies were used: goat anti-mouse IgG Alexa Fluor 488 (1:1000, Molecular Probes), goat anti-rabbit IgG Alexa Fluor 568 (1:1000, Molecular Probes), goat anti-rabbit IgG Alexa Fluor 488 (1:1000, Molecular Probes), goat anti-mouse IgG Alexa Fluor 648 (1:1000, Molecular Probes), goat anti-mouse IgM Alexa Fluor 568 (1:1000, Molecular Probes), goat anti-rat IgM Alexa Fluor 488 (1:1000, Molecular Probes), and goat anti-rabbit IgG Alexa Fluor 648 (1:1000, Molecular Probes).

## Results

### Generation of iPSCs derived from lissencephaly patients and control iPSCs

**Fig. 2:**
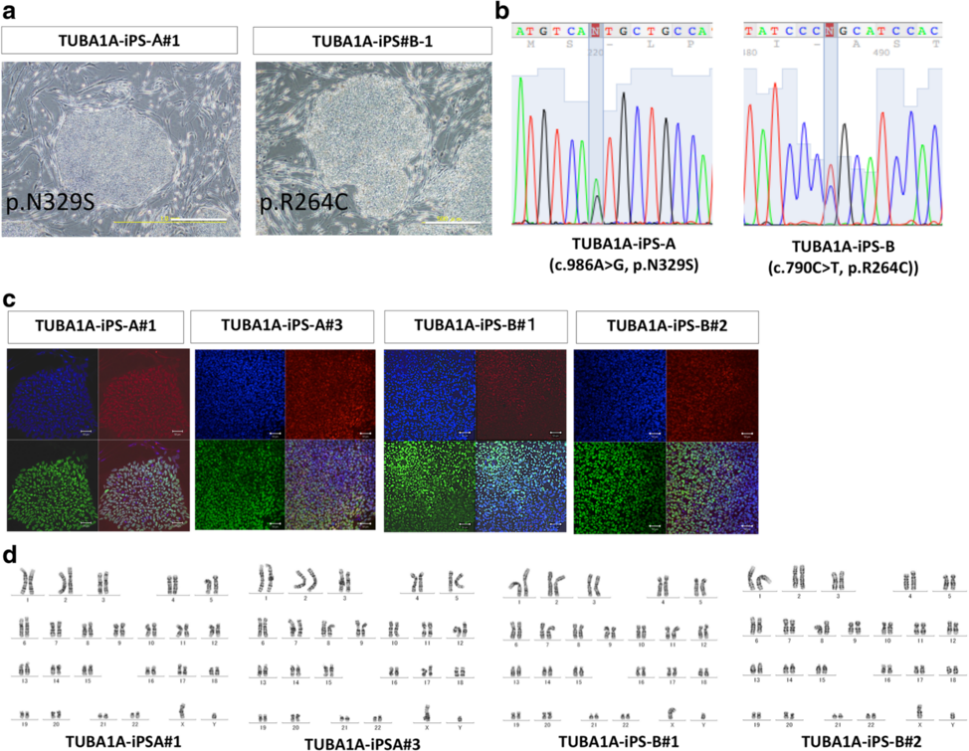
Characterization of control- and patient-derived induced pluripotent stem cells (iPSCs). **a** Phase contrast images of the patient-derived iPSCs (scale bar = 500 μm) showed that both patient-–derived iPSCs had the typical morphology of human pluripotent stem cells on mouse embryonic fibroblast (MEF). **b** Sequencing of α tubulin 1A (*TUBA1A)* in the genomic DNA of the iPSCs showed that the generated iPSCs had missense mutations in *TUBA1A* (c.986A > G in patient A-derived iPSCs, and c.790C > T in patient B-derived iPSCs); these base substitutions cause the p.N329S mutation and p.R264C mutation, respectively, in the TUBA1A protein. **c** Immunocytochemistry for the marker proteins OCT3/4 and NANOG of pluripotent stem cells (scale bar = 50 μm). All the generated iPSCs expressed both OCT3/4 and NANOG. All the iPSCs derived from the lissencephaly patients (TUBA1A-iPS-A#1,3, TUBA1A-iPS-B-#1,2) expressed both OCT3/4 and NANOG. **d** Giemsa staining of the generated iPSCs. Karyotype of the patient-derived iPSCs showed that they had normal 46 XY karyotype

As a first step in generating iPSCs from patients with lissencephaly, gene-transferred UCCs and PBMCs were re-seeded onto the feeder cells of mouse embryonic fibroblasts on day 7 by using nucleofection, and both UCCs and PBMCs were reprogrammed 3 weeks after replating. In the established patient-derived iPSC lines, the colonies appeared like those of human embryonic stem cells (Fig. 2a). *TUBA1A* sequencing showed the heterozygous mutated sequence c.986A > G or c.790C > T (Fig. 2b), and immunocytochemical analyses confirmed the expression of pluripotent marker genes (Fig. 2c). Karyotype analyses showed that these lines maintained a normal karyotype (Fig. 2d). We also established iPSCs (ONH-UCCiPS1#1 from UCC from a healthy volunteer using the same procedure as described above (Additional file 2: Figure S1A).

Quantitative real-time PCR showed that these lines expressed pluripotent cell markers similar to those expressed in the human embryonic stem cells KhES1 (Additional file 2: Figure S1B). These results showed that the newly generated iPSCs differentiated into three germ layers, suggesting that they were pluripotent. PCR analysis of the genomic DNA confirmed that the episomal vector was absent in these iPSCs (Additional file 2: Figure S1C). A teratoma formation assay using subcutaneous transplantation into severely immunodeficient NOG mice was conducted to test for differentiation (Additional file 2: Figure S1D). Collectively, these findings confirmed that the established human iPSC clones in the present study met the general criteria for human iPSCs.

### Generation of iPSC-NPCs using dual SMAD inhibition and neurosphere culture

**Fig. 3:**
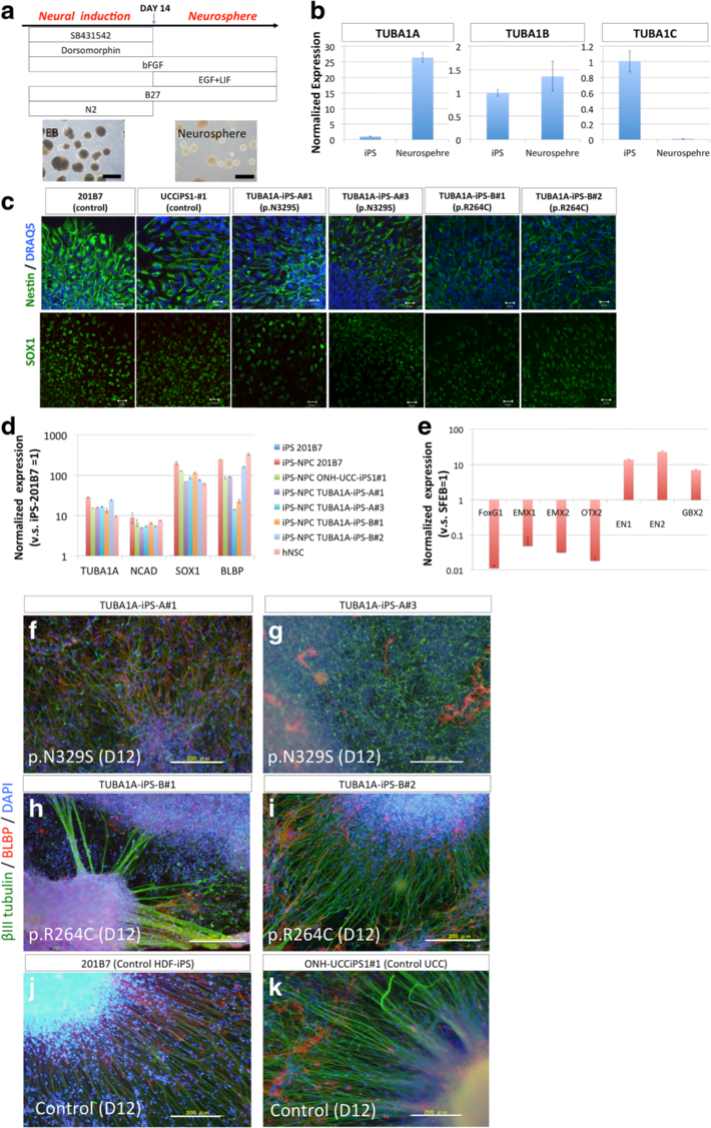
Generation of neurospheres from the induced pluripotent stem cells (iPSCs). **a** Schema of neural induction and neurosphere generation. Neural induction using dual SMAD inhibition and subsequent neurosphere cultures were conducted to obtain the neurospheres. After 2 weeks of neural induction, the embryoid bodies (EBs) were dissociated and cultured in a medium containing bFGF, EGF, and LIF (scale bar = 50 μm). Representative photographs of EBs and neurospheres were derived from Patient A. **b** Quantitative RT-PCR for each *TUBA1* isoform. Through neural induction, only *TUBA1A* expression was greatly increased. Changes in the expression of each *TUBA1* isoform were different (relative to the expression in the undifferentiated iPSCs (201B7), fold change ± SD, *n* = 3, technical duplicates). **c** Immunostaining of each neurosphere (within 12 h after plating in neurosphere medium). Ubiquitous staining for the NPC markers NESTIN and SOX1 (scale bar = 50 μm) was observed in each cell. **d** Quantitative RT-PCR showed that the neurospheres expressed the neural marker genes N-cadherin (*CDH2*) and α tubulin 1A (*TUBA1A*) and the progenitor cell marker *SOX1*, all of which are expressed in the human neural stem cell (hNSC) line. The radial glial marker brain lipid-binding protein (BLBP) was also expressed (relative to the expression in the undifferentiated iPS, fold change ± SD, *n* = 3, technical duplicates). **e** Quantitative RT-PCR showed that the neurospheres had a caudalized regional identity characterized by high gastrulation brain homeobox 2 (*GBX2*) expression and low Forkhead box protein G1 (*FOXG1*) expression (relative to the expression of SFEB used as human forebrain control, fold change ± SD, *n* = 3, technical duplicates). **f**–**k** Spontaneous differentiation of the neurospheres. In 12 days, the βIII tubulin-positive neurites and few BLBP-positive radial glial-like fibers had migrated radially from the neurospheres (scale bar = 200 μm). Poorly ordered neurites and BLBP-positive radial glial-like fibers were observed in the TUBA1A-iPS-A-derived neurospheres (**f**–**g**). Radially extending neurites and BLBP-positive radial glial-like fibers were observed in the TUBA1A-iPS-B-derived neurospheres (H–I) and control iPSC-derived neurospheres (**j**-**k**)

After dual SMAD inhibition, the EBs containing neural progenitor cells were grown for 14 days. Following EB dissociation, proliferative iPSC-NPCs were generated using neurosphere culture with serum-free medium containing bFGF-2, EGF, and LIF (Fig. 3a). Quantitative RT-PCR analysis for *TUBA1A, 1B,* and *1C* showed that only 1A showed remarkable activation during neural induction, suggesting that phenotypic analyses should be performed in the neurospheres and not in the undifferentiated iPSCs (Fig. 3b). The cells in the neurospheres homogeneously expressed the NPC markers SOX1 and NESTIN (Fig. 3c), as confirmed by immunocytochemistry, which indicated that these cells contained NPCs with high purity. Furthermore, quantitative RT-PCR analyses showed that the neural marker *N-Cadherin* and the NPC markers *SOX1* and *BLBP* were highly expressed in each neurosphere (Fig. 3d). Together, these data indicate that iPSC-NPCs could be generated by dual SMAD inhibition and could be proliferated as neurospheres.

*TUBA1A* mutation-associated lissencephaly is characterized by cerebellar hypoplasia and often involves the hypoplasia of the brain stem, including the midbrain and pons. Thus, it is important to determine the regional identity of the neurospheres generated using this method. We therefore performed expression analyses for the region-specific markers.

Interestingly, these propagated neurospheres expressed forkhead box G1 (*FOXG1*; a forebrain marker) at a lower level and gastrulation brain homeobox 2 (*GBX2*; a hindbrain marker) a higher level compared with the miniature brain called “SFEB”, which was used as a human telencephalic control and was generated using previously described methods [19]. This result indicated that these neurospheres belonged to the caudal region, which includes the hindbrain (Fig. 3e).

Soon after differentiation, these neurospheres contained glial, neuronal, and neural progenitor cells, all of which play important roles in fetal CNS development. When the neurospheres were plated, βIII tubulin-positive neuronal cells and several BLBP-positive glial fibers spread toward the outside. BLBP-positive fibers are highly polarized and consist of long processes that extend out of the neurospheres, similar to what was observed for radial glial cells during CNS development (Fig. 3f-k).

At first, we simply compared the morphology of the differentiating neurospheres on Matrigel-coated dishes during early passages (6–8 passages) when the BLBP-positive glial fibers were often observed. From these neurospheres, many neurites extended together with BLBP-positive glial fibers after 12 days (Fig. 3f-k).

Interestingly, the neurites and glial fibers derived from the iPSC-NPCs of the lissencephaly patient A (with the p.N329S *TUBA1A* mutation) did not extend radially and displayed a shorter morphology (Fig. 3f-g). In contrast, the neuronal cells and glial fibers derived from the iPSC-NPCs from patient B carrying the p.R264C *TUBA1A* mutation projected radially (Fig. 3h-i). We believe that these results might be associated with the patient’s pathology. However, quantitative analyses of neuronal morphology was technically challenging due to significant increases in cell density during the 12 days of in vitro differentiation required. Furthermore, undifferentiated NPCs persisted in the center of the neurospheres and continued to produce newborn neurons, which would further complicate quantitative analyses of neuronal morphology such as Sholl analysis.

The number of BLBP-positive glial cells in the neurospheres was low and varied between neurospheres and cell lines, which is consistent with the results of quantitative RT-PCR for *BLBP* (Fig. 3d). To promote the differentiation of NPCs [23] and to reduce variation, establishing cultures with comparable glial content is critical. However, propagation using conditions conducive for neurospheres tends to make the iPSC-NPCs neurogenic rather than gliogenic [24, 25] (Additional file 3: Figure S2), and the precise control of the glial differentiation propensity of the iPSCs remains technically difficult. Therefore, we supplemented the cultures with human glial cells in order to induce robust and rapid differentiation of the iPSC-NPCs.

### Glia-supported neurite extension with fluorescently labeled iPSC-NPCs in neurospheres

**Fig. 4:**
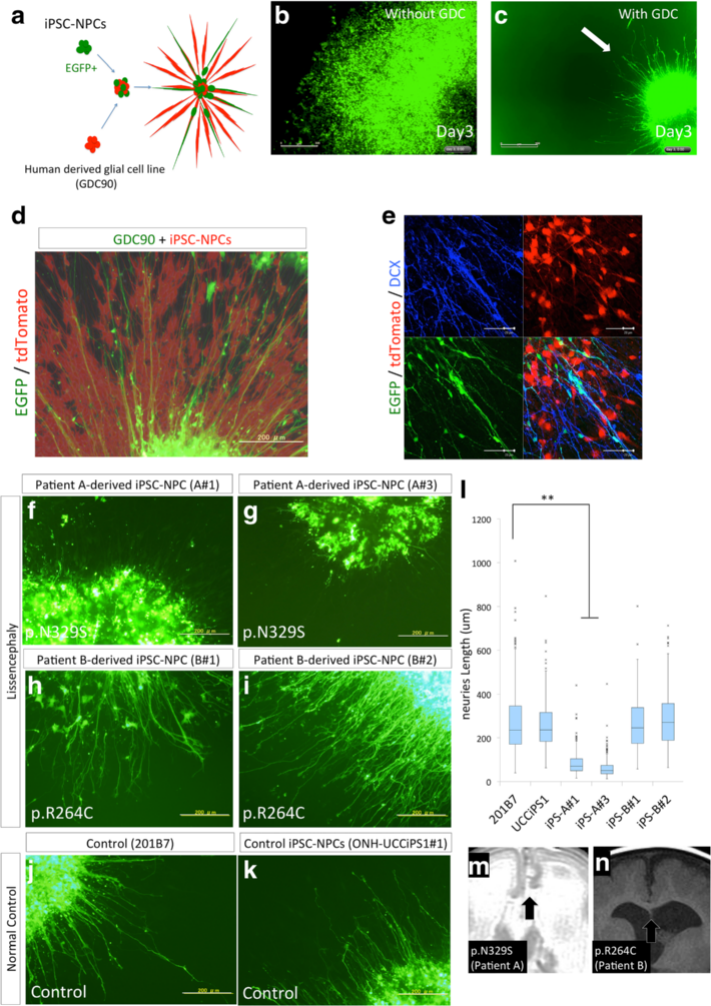
Glia-supported neurite extension method for fluorescently labeled induced pluripotent stem cell (iPSC)-derived neurospheres. **a** Schema of the glia-supported neurite extension method with fluorescently labeled iPSC-derived neurospheres. **b**,**c** Effect of the glia-supported neurite extension method on the fluorescently labeled iPSC-derived neurospheres. After 3 days, the non-polarized cells spread in a disordered manner without a glial cell line; however, using glia-supported neurite extension methods (with GDC90), the differentiating neurites were found to migrate from the neurospheres in a radial and orderly manner (scale bar = 300 μm). Rapid and straightforward extension of the neurites was easily observed using strong EGFP fluorescence. **d** Fluorescence and phase contrast merged images of the glia-supported neurite extension method using EGFP-labeled iPSC-derived neurospheres and tdTomato-labeled human glial GDC90 cells (scale bar = 200 μm). **e** Immunostaining for the young neuron marker doublecortin (DCX) showed that the neurites extending on the tdTomato-positive human glial cells were also stained with EGFP (scale bar = 50 μm). **f**–**k** EGFP fluorescence of the extending neurites observed using the glia-supported neurite extension method on day 5 after plating on the dish. Patient A (p.N329S)-derived neural progenitor cells (NPCs) showed poor neurite extension (**f**–**g**) compared to the Patient B (p.R264C)-derived (**h**-**i**) or healthy control iPSC-derived NPCs (**j**–**k**) (scale bar = 200 μm). **l** Length of EGFP-positive neurites extending from the neurospheres (μm: mean ± SEM). Boxplot graph illustrating median, first quartile, and third quartile with each outliner plotted. (**p < 0.01, one-way ANOVA followed by Dunnett’s test. *n* = 269, 277, 374, 369, 338, and 213 in A#1, A#3, B#1, B#2, 201B7 (control), and UCCiPS1#1, respectively. Statistical analyses were performed using 201B7 as the control. When using ONH-UCCiPS1 independently as a control, the same results were also confirmed.). **m**, **n** Magnified images of coronal scanned MR images showing the morphological differences in the corpus callosum between Patient A (T2-weighted) and Patient B (T1-weighted). The corpus callosum was not present in the MR images (M) of Patient A (with the p.N329S mutation), suggesting that the interhemispheric projection was hypoplastic, unlike that in Patient B (with the p.R264C mutation). Poor neurite extension of patient-derived iPSCs was considered to correspond to the patient’s phenotype and severity of the disease

Genetic labeling with a PiggyBac transposon system was used to establish stable fluorescent iPSC-NPCs to distinguish them from glial cells (Fig. 4a). The iPSC-NPCs were easily propagated by neurosphere culture, enabling the transfection, selection, and propagation of fluorescent cells. The iPSC-NPCs that were co-cultured with the GDC90 cells rapidly polarized and extended their neurites radially compared with those that were grown in the absence of glial cells (Fig. 4b–d and Additional files 4 and 5: Movies S1 and S2). Immunocytochemistry showed that most of the EGFP-positive (iPSC-NPC-derived) neurites expressed DCX, which is a marker for young migrating neurons (Fig. 4e). Time-lapse imaging showed that the EGFP-positive neurites projected radially, and that some of their somas were pulled up from the core of the neurosphere in translocation-like movements in 12 days (Additional files 5 and 6: Movies S2 and S3).

### Analyses of extending neurites using glia-supported neurite extension methods

The behaviors of the patient-derived and control iPSC-NPCs were evaluated using the glia-supported neurite extension method. The measurement of neurite extension after 5 days showed that neurite extension in iPSC-NPCs derived from the patient with the p.N329S *TUBA1A* mutation was severely inhibited (Fig. 4f–g and l, Additional files 7 and 8: Movies S4, S5). These data suggest that neuronal cells that differentiated from the iPSC-NPCs with the p.N329S *TUBA1A* mutation had an immature morphology compared with those derived from the control iPSC-NPCs. We believe that this phenotype corresponded to the pathology of the developing brain because neuronal cells initially polarize from NPCs and migrate toward the cortical plate using various types of neurites, including the leading process and basal process.

This observation was also supported by the results of our experiment using human fetal forebrain-derived NPCs, in which we introduced the CMV early enhancer/chicken beta actin (CAG) promoter-driven p.N329S mutant *TUBA1A* using the PiggyBac transposon based vector system. The overexpression of p.N329S mutant TUBA1A significantly reduced neurite extension of human NPCs (Additional file 9: Figure S3, Additional file 10: Supplementary method), suggesting that lissencephaly related to the p.N329S *TUBA1A* mutation was caused by a dominant-negative effect. Together with the results of our experiments described above, we concluded that we had successfully modeled one aspect of the pathology of lissencephaly *in vitro* using patient-derived iPSC-NPCs with the p.N329S *TUBA1A* mutation.

In contrast, we did not identify any phenotypic difference between the iPSC-NPCs with a p.R264C *TUBA1A* mutation and those derived from the controls (Fig. 4h–k, Additional file 11: Movie S6). One reason for this result may be the disease severity of the patient. The corpus callosum was missing in Patient A (with the N329S mutation), (Fig. 1a-b, Fig. 4m) whereas it is detectable in Patient B (with the p.R264C mutation), as determined from the MR images (Fig. 1c-d, Fig. 4n), suggesting that neurite extension was not as severely affected in Patient B as in Patient A. This finding is consistent with our present *in vitro* results obtained by measuring neurite extension (Fig. 4l). Importantly, this result was also consistent with the gradient in the pathology along the cephalic-caudal axis, as observed in the patients. The brain stem and thalamus, which have a regional identity similar to that of our iPSC-NPCs, did not appear hypoplastic in the MR images obtained from Patient B (Fig. 1d, Table 1).

Overall, the p.N329S *TUBA1A* mutation observed in Patient A showed reduced neurite extension that was caused by mutated tubulin in a dominant-negative manner. In contrast, the neurons generated from the iPSCs from Patient B with the p.R264C *TUBA1A* mutation showed no overt defects in neurite extension. This difference was consistent with the radiological pathology of the TUBA1A lissencephaly patients.

## Discussion

In the present study, we established iPSCs from an easily accessible cell source from patients with lissencephaly and successfully characterized the abnormalities of the iPSC-derived neurons *in vitro*. Recent progress in cell reprogramming methods enabled us to generate iPSCs from a variety of cell sources [9, 11, 26]. For research on human congenital brain malformations, the utility of patient-derived iPSCs overcomes the hurdles that arise owing to the lack of availability of patient-derived cells in extremely rare diseases. Furthermore, we successfully converted these cells into expandable and easy-to-handle iPSC-NPCs using neurosphere culture technology; these resources will be useful in human congenital malformation research in the future.

Lissencephaly is often caused by mutations in microtubule-associated genes [27]. Polarized neuronal cells depend on the microtubule pathway, whereas non-polarized cells depend primarily on the driving force of actin-myosin contraction. To model lissencephaly pathology in the iPSC-NPCs, the dynamic behavior of the polarized neuronal cells must be visualized.

Spontaneously differentiating neurospheres showed extensions of small proportions of BLBP-positive radial fibers and aligned βIII tubulin-positive neurons. In our simple assay, randomly oriented neurons migrated from the neurospheres generated from the iPSCs with the p.N392S *TUBA1A* mutation, which might partially reflect the *in vivo* pathology of the patients with *TUBA1A* mutation-associated lissencephaly. To reduce the variation associated with the glial content of each neurosphere, we used a human glial cell line for robust and rapid neuronal differentiation. Moreover, we could observe the characteristics of the patient-derived neurons themselves without the effects of the endogenous glial cells, which might be susceptible to the effects of mutated TUBA1A or the secondary effects of abnormal neuron-mediated morphological changes that may spoil endogenous glial scaffolds [28]. We also introduced a fluorescent protein into the iPSC-NPCs to observe the behavior of the individual cells using the PiggyBac transposon system. Interestingly, the control iPSC-NPCs in this glial cell scaffold rapidly differentiated into polarized neuronal cells with radially extending neurites. Using this system, we found that the neurite extension from the iPSC-NPCs with the p.N329S *TUBA1A* mutation was inhibited, whereas the iPSC-NPCs carrying the p.R264C mutation showed no apparent abnormality, indicating agenesis of the corpus callosum in Patient A with the N329S mutation. This observation suggested that patient-derived iPSC-NPCs could recapitulate the neurite extension process in the patient brain. Furthermore, neuronal cells with short neurites were also considered immature. Although we have no histological data for these patients, these neurons were considered similar to those of the patient brain because a differentiation defect manifested by immature neurons and persistent radial glia was reported previously in the TUBA1A lissencephaly patient brain [27]. Based on our data from the iPSC-derived NPCs, we concluded that the pathology of p.N329S *TUBA1A* lissencephaly might start from the steps of differentiation or polarization from neural progenitor cells, leading to a migration defect in young neurons and lamination defects in the cerebral cortex. We suspect that p.N329S TUBA1A destabilizes tubulin heterodimer formation by decreasing the number of hydrogen bonds between alpha- and beta-tubulin in the neuronal cells that express abundant TUBA1A for neurite extension [5]. This hypothesis is consistent with the dominant-negative action of p.N329S mutant TUBA1A on neurite extension, determined from the overexpression experiments (Additional file 9: Figure S3). However, considering its effects on the phenotype, the effect of overexpressing the mutant gene would be artificial. Therefore, to investigate the role of N329S, R264C, and other mutations in the disease-related phenotype, further biochemical characterizations, in patient-derived iPSCs and isogenic iPSCs introduced with these mutations by genome editing, is required in our future experiments.

As shown above, we successfully recapitulated one aspect of the pathology of lissencephaly, but the important question of whether neurons derived from a lissencephaly patient can migrate normally has not been answered. The disorganized lamination of the cerebral cortex in lissencephaly patients cannot be easily explained only by the delayed differentiation of neural progenitor cells or the delay in polarization before neuronal migration. To answer this question, further experiments involving the direct observation of migrating human iPSC-derived cortical neurons are needed. We have already generated a miniature brain from iPSCs derived from a patient with severe lissencephaly. However, unexpectedly, we found young cortical neurons located outside the germinal layer (data not shown). The exact mechanism of cortical layer formation in vitro needs to be determined before interpreting this result.

We observed translocation-like movements in this study, which led us to believe that our co-culture system might be useful in the neuronal migration of iPSC-derived telencephalic neurons.

Consistent with the MR images of the lissencephaly patient B, who had a less severe phenotype, iPSC-NPCs with the p.R264C *TUBA1A* mutation showed an almost normal neurite extension, although it is possible that the sensitivity of our assay system was not sufficient to detect the slight change in the phenotype. In contrast to p.N329S TUBA1A, p.R264C TUBA1A was reported to decrease the production of TUBA1A by disturbing protein folding, which might be the reason for the difference in disease severity; that is, the shortage of TUBA1A caused by the p.R264C TUBA1A mutation could be compensated for by other interchangeable α tubulin isoforms via region-specific transcriptional regulation [29–31]. Regional phenotypic differences in *TUBA1A* mutation-associated lissencephaly also might be partially explained by differences in the transcriptional regulation of TUBA1A throughout the brain. Further investigation into TUBA1A regulation is needed to understand the detailed mechanism of TUBA1A-associated lissencephaly.

Finally, we hypothesized that lissencephaly may provide the key to understanding other neurodevelopmental disorders. For example, the pathological analysis of postmortem brain tissue from a patient with autism revealed a partial migration defect [32]. Moreover, the *MeCP2* gene, which is associated with syndromic autism and Rett syndrome, is thought to modulate *TUBA1A* expression [33].

Thus, we believe that an experimental system using iPSC-derived neurospheres from cases of congenital brain malformation would be useful for elucidating the mechanism of the diseases and the development of the human brain.

## Conclusion

We established iPSCs derived from two TUBA1A-associated lissencephaly patients and successfully modeled one aspect of the pathogenesis of lissencephaly in vitro using iPSC-NPCs and iPSC-derived neurons. The disease modeling using iPSCs from patients with brain malformation diseases helped us understand the mechanism underlying rare diseases and human brain development.

## Abbreviation

NPC, neural progenitor cell; iPSC, induced pluripotent stem cells; SFEB, serum-free embyoid body like aggregates; TUBA1A, alpha tubulin 1A; TUBB3, beta III tubulin; GFAP, glial fibrillary acidic protein; BLBP, brain lipid-binding protein; EGFP, enhanced green fluorescent protein; FOXG1, Forkhead box protein G1; GBX2, gastrulation brain homeobox 2

## Additional files

Additional file 1: Table S1. Primer sequences used in this study. * These primers were used in quantitative PCR for pluripotent stem cell markers. (DOCX 19 kb) Additional file 2: Figure S1. (**A**) Characterization of umbilical cord stromal cell-derived control iPSCs (ONH-UCCiPS1#1). ONH-UCCiPS1#1 is morphologically similar to human embryonic stem cells on a mouse embryonic fibroblast feeder. Immunocytochemistry showed that they expressed the pluripotent stem cell markers OCT3/4 and NANOG ubiquitously. (**B**) Quantitative RT-PCR for marker genes of pluripotent stem cells (Relative to the expression in control human embryonic stem cells KhES1, fold change ± SD, *n* = 3, technical duplicates) showed that the generated iPSCs expressed the marker genes. (**C**) Conventional PCR for the detection of persistence of the episomal vector confirmed no major persistence of plasmid vectors (EP4 stands for the primer set designed using detection of the episomal vector-specific sequence). (**D**) Teratoma formation assay using subcutaneous transplantation in NOD/Shi-scid IL2Rg null (NOG) mice (scale bar = 100 μm) showed that the patient-derived iPSCs could differentiate into three germ layers. (TIF 19759 kb) Additional file 3: Figure S2. (**A**-**C**) Immunocytochemistry for the immature neuronal marker βIII tubulin and glial cell marker GFAP. Most of the dissociated iPSC-NPCs were differentiated into neurons in serum-free differentiation medium within 2 weeks. (**D**) Quantification of data from immunocytochemical analyses showed that the dissociated iPSC-NPCs were differentiated predominantly (>90 %) into βIII tubulin neurons in 2 weeks. GFAP-positive cells were very rarely (<1 %) observed in our differentiation condition (mean ± SD, *n* = 3 technical duplicates of each cell line, control; 201B7 and UCCiPS1#1, p.N329S; TUBA1A-iPS-A#1, A#3, p.R264C; TUBA1A-iPS-B#1, B#2). No significant differences in the neurogenicity and gliogenicity were observed between these cells (Welch’s *t* test). (**E**) Quantitative RT-PCR showed that the expression of the glial marker GFAP in the differentiated iPSC-NPCs was extremely low (mean ± SD, *n* = 3 technical duplicates of each cell line, control; 201B7 and UCCiPS1#1, p.N329S; TUBA1A-iPS-A#1, A#3, p.R264C; TUBA1A-iPS-B#1, B#2). This result was consistent with the data obtained from the immunocytochemical analyses shown in Additional file 3: Figure S2A. In addition, there were no significant differences in GFAP expression between these cell lines (Welch’s *t* test). (**F**) Quantitative PCR showed that the immature neuronal marker TUBB3 in the differentiated iPSC-NPCs was expressed in all the cell lines, and that the differences were not statistically significant (Welch’s *t* test, mean ± SD, *n* = 3 technical duplicates of each cell line, control; 201B7 and UCCiPS1#1, p.N329S; TUBA1A-iPS-A#1, A#3, p.R264C; TUBA1A-iPS-B#1, B#2). (TIF 19759 kb) Additional file 4: Movie S1. Fluorescent neurosphere (201B7) differentiation without glial cells. (AVI 25686 kb) Additional file 5: Movie S2. Fluorescent neurosphere (201B7) differentiation with glial cells. (AVI 30161 kb) Additional file 6: Movie S3. Translocation-like movement in differentiating fluorescent neurospheres (201B7) at high magnification. (AVI 2472 kb) Additional file 7: Movie S4. Neurite extension (201B7) with the glia-supported neurites extension method. (AVI 1390 kb) Additional file 8: Movie S5. Neurite extension (iPS-A) with the glia-supported neurites extension method. (AVI 891 kb) Additional file 9: Figure S3. Differentiating pPBCAG-TUBA1A-IRES-AcGFP-transfected human NPCs. Measurements of AcGFP-positive neurites from each cell (μm: mean ± SEM, *n* = 50, one-way ANOVA followed by Dunnett’s test, ***p* < 0.01). Overexpression of mutant *TUBA1A (p.N329S)* interfered with neurite extension in the human NSCs (scale bar = 200 μm). (TIF 19759 kb) Additional file 10: Supplementary method. (DOCX 72 kb) Additional file 11: Movie S6. Neurite extension (iPS-B) with the glia-supported neurites extension method. (AVI 1492 kb)
