# Supplementary material for: In vitro characterization of neurite extension using induced pluripotent stem cells derived from lissencephaly patients with TUBA1A missense mutations
Source: Mol Brain. 2016 Jul 19;9:70. doi: 10.1186/s13041-016-0246-y (PMC4950778; doi:10.1186/s13041-016-0246-y)
Supplement: Additional file 1: Table S1. — Primer sequences used in this study. * These primers were used in quantitative PCR for pluripotent stem cell markers. (DOCX 19 kb) [file 13041_2016_246_MOESM1_ESM.docx]

Supplementary Table 1.

| Primer | Use | Sequence (5' to 3') |
| --- | --- | --- |
| pEP4-SR | PCR | TCGGGGGTGTTAGAGACAAC |
| pEP4-SF | PCR | TTCCACGAGGGTAGTGAACC |
| GAPDH-F | PCR | ACGACCCCTTCATTGACCTCAACT |
| GAPDH-R | PCR | ATATTTCTCGTGGTTCACACCCAT |
| TUBA3-4AF | Genotyping | AGAAAGCTGTTCATGGTAGG |
| TUBA3-4AR | Genotyping | TCCTGGAAGATGTATGAAAAG |
| TUBA3-4BF | Genotyping | CAGACCACAACTTTTCAATG |
| TUBA3-4BR | Genotyping | CCCTGAATGTTGACCTGAC |
| TUBA1A-cDNA-seq-F | RT-PCR  Sequencing | CTGAATAGGTTAATAGGTCAAATTGTGTC |
| TUBA1A-cDNA-seq-F | RT-PCR  Sequencing | CTCTGTACCTTGGCCAGGTC |
| GAPDH-F | qPCR | CGCTCTCTGCTCCTCCTGTT |
| GAPDH-R | qPCR | CCATGGTGTCTGAGCGATGT |
| NESTIN-F | qPCR | CCAAGACTGCCCTGGAAAC |
| NESTIN-R | qPCR | CCTCCCTCTCCAAGGAAACA |
| PAX6-F | qPCR | GTGTCTACCAACCAATTCCACAAC |
| PAX6-R | qPCR | CCCAACATGGAGCCAGATG |
| BLBP-F | qPCR | GGACTCTCAGCACATTCAAGAA |
| BLBP-R | qPCR | CCACATCACCAAAAGTAAGGGT |
| FOXG1-F | qPCR | GCCACAATCTGTCCCTCAACA |
| FOXG1-R | qPCR | CGGGTCCAGCATCCAGTAG |
| TBR1-F | qPCR | ATGGGCAGATGGTGGTTTTA |
| TBR1-R | qPCR | GACGGCGATGAACTGAGTCT |
| TBR2-F | qPCR | CACCGCCACCAAACTGAGAT |
| TBR2-R | qPCR | CGAACACATTGTAGTGGGCAG |
| EMX1-F | qPCR | AGGTGAAGGTGTGGTTCCAG |
| EMX1-R | qPCR | AGTCATTGGAGGTGACATCG |
| EMX2-F | qPCR | GCTTCTAAGGCTGGAACACG |
| EMX2-R | qPCR | CCAGCTTCTGCCTTTTGAAC |
| OTX-2-F | qPCR | ATCTTCATGCGAGAGGAGGTG |
| OTX-2-R | qPCR | CATTCTGCTGTTGTTGCTGTTG |
| EN1-F | qPCR | TGGGTGTACTGCACACGTTTTC |
| EN1-R | qPCR | TGTCCTCCTTCTCGTTCTTCTTCT |
| EN2-F | qPCR | CTCGGACTCGGACAGCTC |
| EN2-R | qPCR | CTTTGGTTTTCGAGACCTGG |
| GBX2-F | qPCR | GGTGCAGGTGAAAATCTGGT |
| GBX2-R | qPCR | CCTGTCTTGGAATTGGCATT |
| CTIP2-F | qPCR | GAGTACTGCGGCAAGGTGTT |
| CTIP2-R | qPCR | TAGTTGCACAGCTCGCACTT |
| VGLUT1-F | qPCR | GAAACTCATGAACCCCCTCA |
| VGLUT1-R | qPCR | GGGAGATGAGCAGCAGGTAG |
| VGLUT2-F | qPCR | ATTCCATCAGCAGCCAGAGT |
| VGLUT2-R | qPCR | TTGCTCCATATCCCATGACA |
| SOX1-F | qPCR | GCAAGATGGCCCAGGAGAA |
| SOX1-R | qPCR | CCTCGGACATGACCTTCCA |
| NCAD-F | qPCR | GGACAGTTCCTGAGGGATCA |
| NCAD-R | qPCR | GGATTGCCTTCCATGTCTGT |
| TUBA1A-F | qPCR | AGCTCGGCAGTCGCGAAGCAG |
| TUBA1B-F | qPCR | CTTCGCCTCCTAATCCCTAGCCAC |
| TUBA1C-F | qPCR | CCGCAGACCCCTTCAAGTTCTAGTC |
| TUBA1-R | qPCR | CAATGACTGTGGGTTCCAAGTCTAC |
| Oct3/4-F | qPCR | GACAGGGGGAGGGGAGGAGCTAGG |
| Oct3/4-R | qPCR | CTTCCCTCCAACCAGTTGCCCCAAAC |
| GAPDH-F | qPCR* | CCACTTTGTCAAGCTCATTTCCT |
| GAPDH-R | qPCR* | TCTCTTCCTCTTGTGCTCTTGCT |
| NANOG-F | qPCR | GCAGAAGGCCTCAGCACCTA |
| NANOG-R | qPCR | GGTTCCCAGTCGGGTTCAC |
| SOX2-F | qPCR | GGGAAATGGGGAGGGGTGCAAAAGAGG |
| SOX2-R | qPCR | TTGCGTGAGTGTGGATGGGATTGGTG |
| ZFP42-F | qPCR | CAGATCCTAAACAGCTCGCAGAAT |
| ZFP42-R | qPCR | GCGTACGCAAATTAAAGTCCAGA |
| DNMT3B-F | qPCR | TGCTGCTCACAGGGCCCGATACTTC |
| DNMT3B-R | qPCR | TCCTTTCGAGCTCAGTGCACCACAAAAC |
| TUBB3-F | qPCR | GGGCCTTTGGACATCTCTTC |
| TUBB3-R | qPCR | ACTCCTTCCGCACCACATC |
| GFAP-F | qPCR | ACATCGAGATCGCCACCTAC |
| GFAP-R | qPCR | ACATCACATCCTTGTGCTCC |

* These primers were used in quantitative PCR for pluripotent stem cell marke
