# Supplementary figures and images for: In vitro characterization of neurite extension using induced pluripotent stem cells derived from lissencephaly patients with TUBA1A missense mutations
Source: Mol Brain. 2016 Jul 19;9:70. doi: 10.1186/s13041-016-0246-y (PMC4950778; doi:10.1186/s13041-016-0246-y)

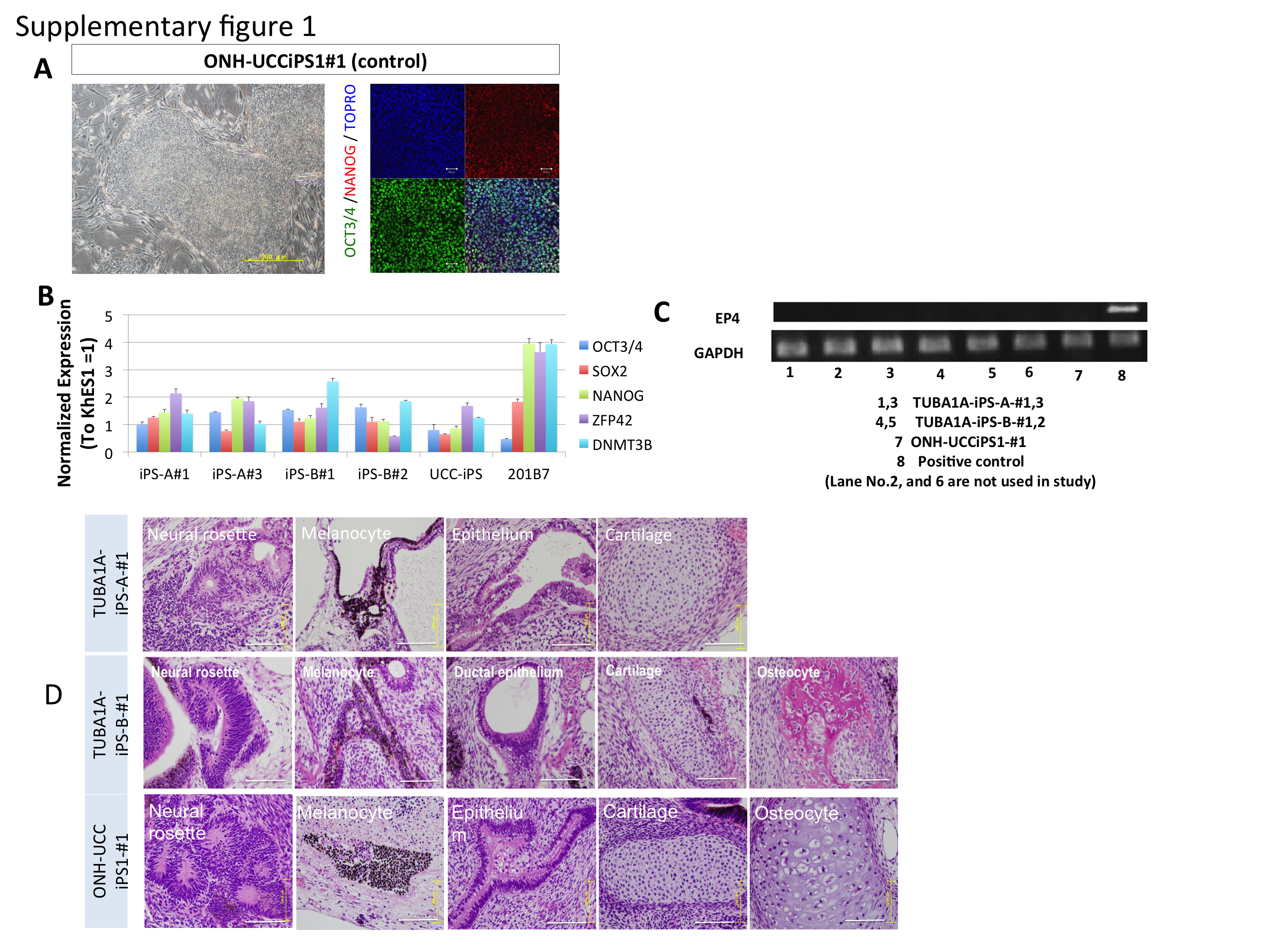

Supplement: Additional file 2: Figure S1. — (A) Characterization of umbilical cord stromal cell-derived control iPSCs (ONH-UCCiPS1#1). ONH-UCCiPS1#1 is morphologically similar to human embryonic stem cells on a mouse embryonic fibroblast feeder. Immunocytochemistry showed that they expressed the pluripotent stem cell markers OCT3/4 and NANOG ubiquitously. (B) Quantitative RT-PCR for marker genes of pluripotent stem cells (Relative to the expression in control human embryonic stem cells KhES1, fold change ± SD, n = 3, technical duplicates) showed that the generated iPSCs expressed the marker genes. (C) Conventional PCR for the detection of persistence of the episomal vector confirmed no major persistence of plasmid vectors (EP4 stands for the primer set designed using detection of the episomal vector-specific sequence). (D) Teratoma formation assay using subcutaneous transplantation in NOD/Shi-scid IL2Rg null (NOG) mice (scale bar = 100 μm) showed that the patient-derived iPSCs could differentiate into three germ layers. (TIF 19759 kb) [file 13041_2016_246_MOESM2_ESM.tif]

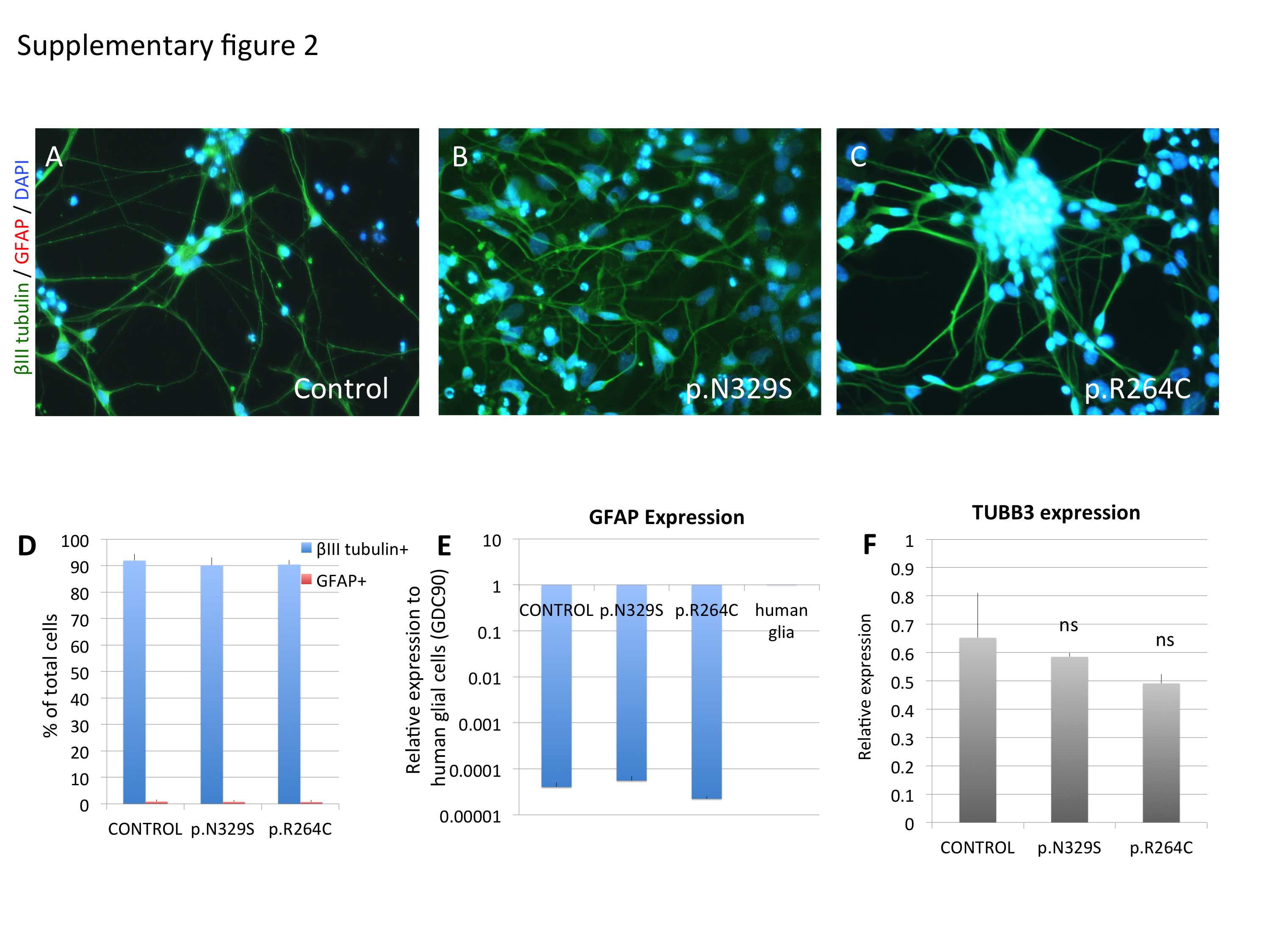

Supplement: Additional file 3: Figure S2. — (A-C) Immunocytochemistry for the immature neuronal marker βIII tubulin and glial cell marker GFAP. Most of the dissociated iPSC-NPCs were differentiated into neurons in serum-free differentiation medium within 2 weeks. (D) Quantification of data from immunocytochemical analyses showed that the dissociated iPSC-NPCs were differentiated predominantly (>90 %) into βIII tubulin neurons in 2 weeks. GFAP-positive cells were very rarely (<1 %) observed in our differentiation condition (mean ± SD, n = 3 technical duplicates of each cell line, control; 201B7 and UCCiPS1#1, p.N329S; TUBA1A-iPS-A#1, A#3, p.R264C; TUBA1A-iPS-B#1, B#2). No significant differences in the neurogenicity and gliogenicity were observed between these cells (Welch’s t test). (E) Quantitative RT-PCR showed that the expression of the glial marker GFAP in the differentiated iPSC-NPCs was extremely low (mean ± SD, n = 3 technical duplicates of each cell line, control; 201B7 and UCCiPS1#1, p.N329S; TUBA1A-iPS-A#1, A#3, p.R264C; TUBA1A-iPS-B#1, B#2). This result was consistent with the data obtained from the immunocytochemical analyses shown in Additional file 3: Figure S2A. In addition, there were no significant differences in GFAP expression between these cell lines (Welch’s t test). (F) Quantitative PCR showed that the immature neuronal marker TUBB3 in the differentiated iPSC-NPCs was expressed in all the cell lines, and that the differences were not statistically significant (Welch’s t test, mean ± SD, n = 3 technical duplicates of each cell line, control; 201B7 and UCCiPS1#1, p.N329S; TUBA1A-iPS-A#1, A#3, p.R264C; TUBA1A-iPS-B#1, B#2). (TIF 19759 kb) [file 13041_2016_246_MOESM3_ESM.tif]

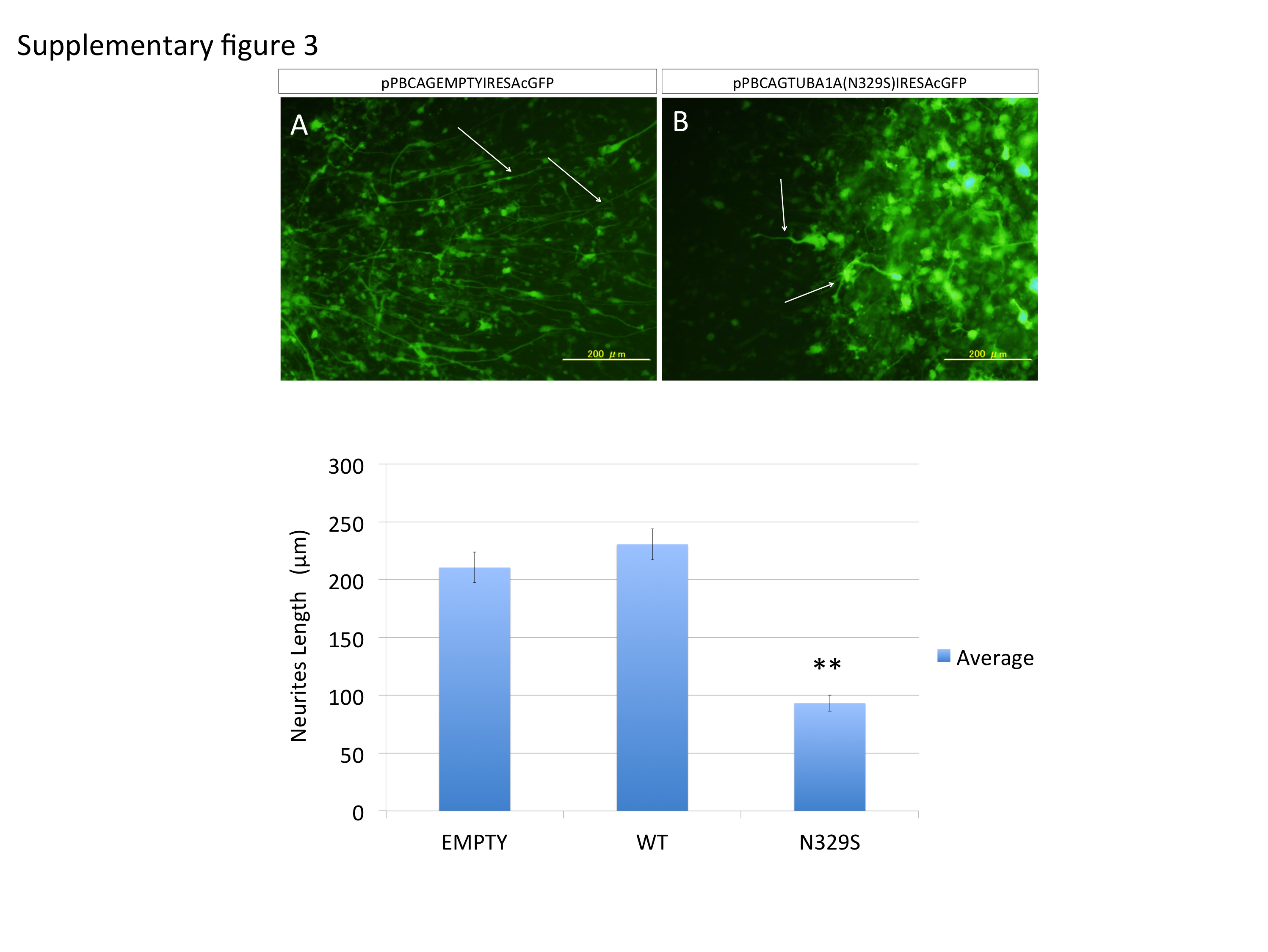

Supplement: Additional file 9: Figure S3. — Differentiating pPBCAG-TUBA1A-IRES-AcGFP-transfected human NPCs. Measurements of AcGFP-positive neurites from each cell (μm: mean ± SEM, n = 50, one-way ANOVA followed by Dunnett’s test, **p < 0.01). Overexpression of mutant TUBA1A (p.N329S) interfered with neurite extension in the human NSCs (scale bar = 200 μm). (TIF 19759 kb) [file 13041_2016_246_MOESM9_ESM.tif]
