# Supplementary material for: In vitro characterization of neurite extension using induced pluripotent stem cells derived from lissencephaly patients with TUBA1A missense mutations
Source: Mol Brain. 2016 Jul 19;9:70. doi: 10.1186/s13041-016-0246-y (PMC4950778; doi:10.1186/s13041-016-0246-y)
Supplement: Additional file 10: — Supplementary method. (DOCX 72 kb) [file 13041_2016_246_MOESM10_ESM.docx]

**SUPPLEMENTARY METHODS**

***Effect of mutant tubulin overexpression in a human neural stem cell line***

Expression vectors were constructed from the backbone of a PB533A (System BioSciences, Laurie Goldman, CA, USA), a CAG promoter, and a neomycin-resistance cassette of pEB multi-neo (Wako) and the IRES-AcGFP cassette (Takara Bio, Shiga, Japan), resulting in pPBCAGIAneo. Untagged *TUBA1A* cDNA (Origene, Rockville, MD, USA) was cloned into the XhoI site of pPBCAGIAneo using an XhoI linker (Takara Bio). Mutated cDNA was generated using a PrimeStar Mutagenesis Kit (Takara Bio) with a primer set designed according to the manufacturer’s instructions, and its sequences were confirmed before cloning into an expression vector. Expression PiggyBac vectors were transfected into a human fetal forebrain-derived neural stem cell line (oh-NSC-3-fb) [17]. Transfected neurospheres were plated on Matrigel-coated glass-bottom dishes. The radially extending leading processes were detected by fluorescence, and their lengths were calculated using ImageJ software and statistically analyzed using one-way ANOVA followed by Dunnett’s test.
